# A high-resolution gridded dataset to assess electrification in sub-Saharan Africa

**DOI:** 10.1038/s41597-019-0122-6

**Published:** 2019-07-03

**Authors:** Giacomo Falchetta, Shonali Pachauri, Simon Parkinson, Edward Byers

**Affiliations:** 1 Energy Program, International Institute for Applied Systems Analysis (IIASA), Schossplatz 1, 2361 Laxenburg, Austria; 2 Future Energy Program, Fondazione Eni Enrico Mattei (FEEM), Corso Magenta 63, 20123 Milan, Italy; 3 Institute for Integrated Energy Systems, University of Victoria, PO BOX 3055 STN CSC, Victoria, Canada

**Keywords:** Energy supply and demand, Developing world, Energy access

## Abstract

Spatially explicit data on electricity access and use are essential for effective policy-making and infrastructure planning in low-income, data-scarce regions. We present and validate a 1-*km* resolution electricity access dataset covering sub-Saharan Africa built on gridded nighttime light, population, and land cover data. Using light radiance probability distributions, we define electricity consumption tiers for urban and rural areas and estimate the by-tier split of consumers living in electrified areas. The approach provides new insight into the spatial distribution and temporal evolution of electricity access, and a measure of its quality beyond binary access. We find our estimates to be broadly consistent with recently published province- and national-level statistics. Moreover, we demonstrate consistency between the estimated electricity access quality indicators and survey-based consumption levels defined in accordance with the World Bank Multi-Tier Framework. The dataset is readily reproduced and updated using an open-access scientific computing framework. The data and approach can be applied for improving the assessment of least-cost electrification options, and examining links between electricity access and other sustainable development objectives.

## Abstract (summary)

**Table:** 

Design Type(s)	modeling and simulation objective • observational design • data integration objective
Measurement Type(s)	Electricity
Technology Type(s)	digital curation
Factor Type(s)	temporal_interval • geographic location
Sample Characteristic(s)	Sub-Saharan Africa • anthropogenic habitat

Machine-accessible metadata file describing the reported data (ISA-Tab format)

## Background & Summary

The United Nations’ Sustainable Development Goal 7 (SDG 7) aims at ensuring access to affordable, reliable, sustainable and modern energy for all by 2030^1^. Access to electricity services (SDG targets 7.1.1 and 7.A and 7.B) is a key priority under this goal - also due to the strong interconnections it exhibits with other development objectives^2–5^, but achieving it globally presents significant challenges^6^. Spatially-explicit, up-to-date and harmonized information on electrification progress is needed to identify where local, national and international efforts can be focused to achieve the most benefits.

Tracking initiatives such as the *Progress Towards Sustainable Energy* portal^7^, the *Status of Electricity Access Report*^8^, and the publication of field-collected data^9,10^ have quantified electrification progress over the last 20 years. It is estimated that in 2016 the global population without access dipped below 1 billion^11^, largely as a result of international investment flows^12^, declining costs of distributed access solutions (e.g., solar PV)^13^, and supporting policy frameworks^14^. Of those still without access, around 600 million live in sub-Saharan Africa (SSA), where 15 countries have electrification levels below 25%^11^.

Monitoring at national scales is important to track efforts regularly. Yet, assessing sub-national heterogeneity is essential to render a clearer picture of the electrification status of a country and improve the efficacy, design, and implementation of local-scale electrification plans^15,16^. This is because electrification efforts could be concentrated in specific regions, such as those nearer to existing grid-connections, whereas progress may remain slow or even stagnate in more remote areas that lack access to energy resources^17,18^. Moreover, data on differences in consumption levels are needed to assess the quality and inequality of access and subsequent implications of these access inequalities^19^. However, actual electricity consumption data are rarely available at a high level of spatial resolution. Using field-surveys to collect such data are costly and time-intensive. In this context, an ongoing debate is centered on how to best deliver good quality data to track energy access objectives and to lower its collection and processing costs, as well as to make sound decisions under data scarcity^20^.

Here, we present and validate an updatable, fully-reproducible 1-*km* resolution dataset of electricity access and use tiers for SSA built on regularly updated gridded nighttime light (NTL), population, and land cover data. The dataset is in the form of netCDF files with counts of the number of people without access in each 1-*km*^2^ cell for each year between 2014 and 2018 and the estimated tier of consumption in each pixel for 2018. The estimates for each metric and country are validated with the most recent available figures. The data can be browsed interactively in a web-based interface. The entire code - from data generation, processing, and export - is available for results reproduction. New NTL composites are published each month, allowing continuous updates. Our estimates are broadly consistent with both field survey-based province-level electrification levels reported by DHS surveys, and aggregated national-level figures published yearly by the the ESMAP/World Bank *Tracking SDG 7* report. The dataset can be combined with other regional-level data and implemented in an array of studies for developing scenarios and modelling pathways of energy access and demand in Integrated Assessment Models^21,22^, assessing least-cost electrification options^15^, tracking development objectives at a local scale, designing infrastructure, and analysing links between electricity access and other socio-economic and well-being metrics.

**Fig. 1:**
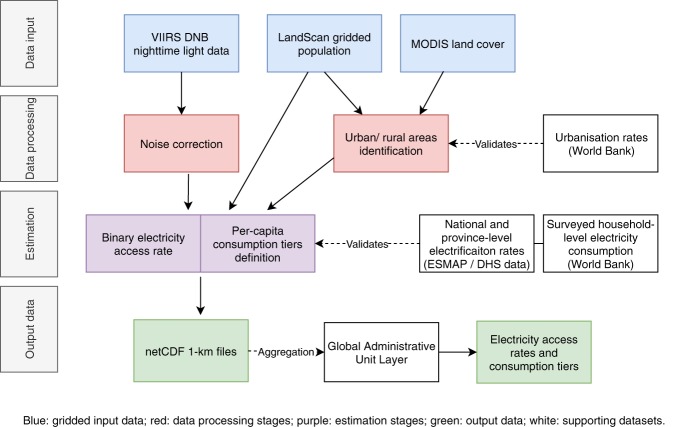
Schematic of the open-source scientific computing framework. The flowchart represents the different stages, data inputs, and outputs in the generation and validation of the data. Blue: remotely-sensed variables; red: processing steps; purple: output metrics; green: output data; white: validation and supporting datasets; grey: stages.

Figure 1 illustrates a schematic of the open-source scientific computing framework, which is discussed in detail in the Methods section. Recent high-resolution data assembling efforts for nighttime light (NTL)^23^, population^24,25^, and land cover^26^, and cloud computing facilities for spatial data processing^27^ enable estimating the evolution in electricity access and consumption tiers at the 1-*km*^2^-pixel-level. We employ VIIRS-DNB (*Visible Infrared Imaging Radiometer Suite, Day-Night Band*) stray-light corrected product for NTL radiance (450 *m* resolution at the equator) and LandScan (used as the main source) and WorldPop (for comparison) gridded population distribution datasets, both with a 1-*km* resolution. WorldPop was only used as a secondary source due to possible circularity issues since the underlying model also uses NTL as an input for estimating population. Refer to https://www.popgrid.org/compare-data for a comparative overview of inputs and methodology of different gridded population datasets. The use of Landscan and WorldPop supports accounting for recent population growth (on average 4.1% in urban areas vs. 2.7% for SSA as a whole in 2017, according to World Bank Data, https://data.worldbank.org/) and migration dynamics.

Nighttime lights have already been used as a proxy for electricity access^28–31^, residential consumption^32–37^, outages detection^38–40^, population fluctuations and migration^41,42^, regional GDP^43,44^ and income inequality^45^. This is, however, the first time a fully reproducible and updatable dataset at this high level of resolution has been constructed and validated for SSA.

## Methods

### Data sources and processing tools

The data sources - described in Table 1 - include VIIRS-DNB stray-light corrected NTL monthly composites for the years 2014–2018^23^ (which measure radiance and filter cloud cover), LandScan 2014 and 2017 downscaled gridded population^24^, and WorldPop 2015 and 2020^25^ for sensitivity analysis. National electricity access rates are drawn from the ESMAP/World Bank tracking SDG 7 portal^7^, while province-level figures are drawn from an array of field surveys through the USAID/DHS Program STATcompiler for sub-national benchmarking^10^. Household survey data to validate consumption tiers have been retrieved from the WorldBank Microdata Library (http://microdata.worldbank.org). For defining countries, we adopted the global administrative boundaries (GADM) dataset v3.6^46^.

**Table 1:** Table of data inputs for electrification data generation and validation.

Dataset	Source	Time resolution	Spatial resolution	Year(s)
VIIRS-DNB nighttime lights	^23^	1 month	0.008°	2014–2018
LandScan gridded population	^24^	1 year	1 km	2014–2017
WorldPop gridded population	^25^	1 year	1 km	2015 and 2020
MODIS Land Cover Type, University of Maryland classification	^26^	1 year	450 m	2017
GADM global administrative layers	^46^	—	—	2018
USAID/DHS StatCompiler surveys	^10^	1–2 years	Province-level	2014–2018, depending on country
ESMAP/World Bank electrification database	^7^	1 year	Country-level	2014–2016
World Bank MicroData Library household surveys	—	1–2 years	Household-level	2014–2018, depending on country

The Google Earth Engine platform^27^ is used to process satellite imagery and extract data, which is subsequently used to produce the datasets and generate plots in the R scientific computing environment. The Code Availability and Data Records section links to the repositories that host the Earth Engine Javascript code and R script allowing for results reproduction, alteration of parameters for further sensitivity analysis, or other improvements as implemented by new users. Results are also dynamically visualized in a web interface, under https://gdessa.ene.iiasa.ac.at.

### Urban and rural areas identification

The definition of urbanisation is highly variable across countries and regions of both Africa^47,48^ and the entire world^28^. The United Nations’ definition of urban and rural population is tightly linked to political and administrative factors that make it challenging to generalize the human settlement structure across different countries^49^. Recently, new methods have been promoted to overcome such issues, especially in high-income countries^50^. Those rely on satellite imagery and land cover, but also on functional definitions, e.g. accounting for the entire commuting zones surrounding cities.

For identifying urban and rural settlements at the grid-cell level, we adopt the 2017 MCD12Q1 V6 MODIS Land Cover Type^26^ from the Annual University of Maryland classification (having a resolution of 500 *m*) in combination with the 2017 LandScan population (1-*km* resolution). Both datasets are updated on an annual basis. For urban areas, land cover type 13 (urban and built-up lands) is used as a necessary condition, in combination with a population density higher than specific *inhab* · *km*^−2^ thresholds.

Supplementary Table 1 lists the clusters of countries and the corresponding thresholds which have been defined and applied so as to minimize the discrepancy with World Bank reported urban-rural population split for 2017 (https://data.worldbank.org/). Threshold bands partially overlap with geographic regions, such as Southern Africa, where a threshold of more than 650 *inhab* · *km*^−2^ is found to effectively predict urban areas. Nonetheless, for some countries their threshold does not match their region and they were inserted in density clusters which allowed a better estimation of their reported urbanization rate. For rural settlements, land area with a population density lower than that of each cluster’s threshold was considered. Non-populated land was discarded.

The approach results in a very consistent estimate (*R*^2^ = 0.84 and 0.067 residual standard error in a simple linear regression) vis-Ã -vis the World Bank/UN population division figures built on countries’ official national statistics. Supplementary Fig. 1 shows the prediction obtained with the approach.

### Electricity access estimation

**Fig. 2:**
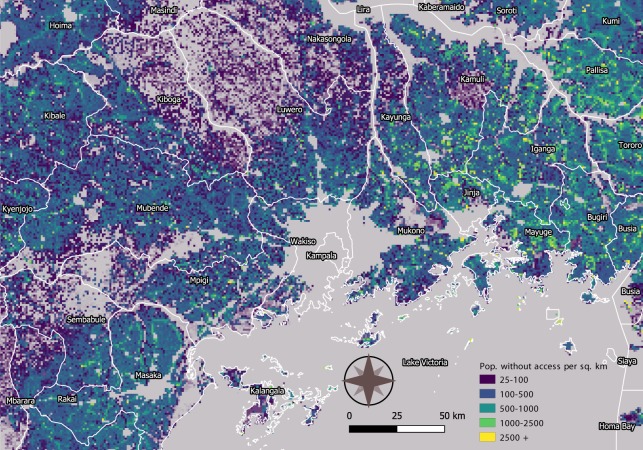
Distribution of people without access over Uganda in 2018. The figure provides a sample representation of the output dataset. Colours represent the density of people without electricity access in each 1-*km* pixel. Administrative boundaries correspond to the first level of the GADM definition^46^.

A large body of literature has shown that locations where satellite sensors cannot detect visible light are likely to be areas where people live without access to electricity^28–30,51^. We calculated the median value of radiance within each pixel of the NPP-VIIRS monthly composites for both 2014 and 2018 within the Google Earth Engine Platform; a lower-bound noise floor was set at 0.25 *μW* · *cm*^−2^ · *sr*^−1^ for 2014 and to 0.35 *μW* · *cm*^−2^ · *sr*^−1^ for 2018 to remove calibration noise and ephemeral lights as discussed in the relevant literature^52–55^. The discrepancy in the noise caps is justified on the grounds of the increased noisiness of the data witnessed from 2017. According to expert opinion, this is owing to calibration adjustments in the lunar correction and to the launch of a new VIIRS-equipped satellite (JPSS J1) in 2017 (as discussed in^56^). The spread in the noise lower-bound cap set before and after 2017 was determined from the examination of by-definition zero radiance pixels - such as within large water bodies - which nonetheless present a systematically positive radiance for data since 2017, and a systematically zero radiance for previous data. Refer to the Supplementary Information for histograms comparing the distribution of background noise before and after 2017 for the same non-populated area. Population counts within each GADM level 1 provinces have been produced for both people living in lit (above 0.25/0.35 *μW* · *cm*^−2^ · *sr*^−1^) and in dark areas (≤0.25/0.35 *μW* · *cm*^−2^ · *sr*^−1^). The gridded 1-*km* classification was used to aggregate gridded results into spatial polygon features representing provincial and national administrative boundaries. Aggregation of the gridded data is achieved assuming grid cells belong to the polygon that contain its centroid. To provide an example of the output data, Fig. 2 shows a snapshot of the estimated density of people without electricity access over Uganda in 2018.

### Classification of consumption tiers

Previous studies have shown that residential electricity consumption can be accurately predicted with satellite derived NTL data at both the country^57^ and the province-level^58^. Here, we considered a measure of radiance for those estimated to live in areas with electricity access. Based on the distribution of the quartile values of non-zero light radiance across SSA countries (Supplementary Fig. 2), four tiers of residential electricity consumption were defined, with thresholds set at the median value of each quartile distribution. In order to account for the strong urban-rural discontinuity in lighting, this was done separately for urban and rural settlements. Settlement classification - and thus indirectly also tiers - is based on population density data and accounts for the heterogeneity in densities across countries, as described previously. Corresponding tier thresholds are reported in Table 2.

**Table 2:** Definition of radiance tiers (*μW* · *cm*^−2^ · *sr*^−1^) for electrified areas used to estimate consumption levels.

Tier	Urban		Rural	
	Lower-bound	Upper-bound	Lower-bound	Upper-bound
1	>0	<0.40	>0	<0.38
2	≥0.40	<0.48	>0.38	<0.45
3	≥0.48	<0.88	>0.45	<0.68
4	≥0.88	—	>0.68	—

The four tiers are validated against survey data consistent with the World Bank Multi-Tier framework^59^, as discussed in the Technical Validation Section. The tier data can be mapped onto a gridded population dataset to derive the share of population with access to electricity in each consumption tier in both urban and rural areas.

## Data Records

The data^60^ are hosted on Mendeley Data at the permanent 10.17632/kn4636mtvg. The repository includes two netCDF v4 files containing the data output of the analysis, which are reported in Table 3. The first file reports the number of people without access in each grid cell between 2014 and 2018. The second reports the consumption tier in each populated, electrified cell for the year 2018. Both files have a 1-km spatial resolution and cover sub-Saharan Africa entirely. The data may be shared, used and adapted even for commercial use with the requirement of attributing to this original work as per a Creative Commons BY v4.0 (CC BY 4.0) License.

**Table 3:** Output data files, resolution, and format.

File	Time resolution	Spatial resolution	Format
Density of people without electricity access	1 year (2014–2018)	1 km	netCDF v4
Tier of electricity consumption	1 year (2018)	1 km	netCDF v4

## Technical Validation

**Fig. 3:**
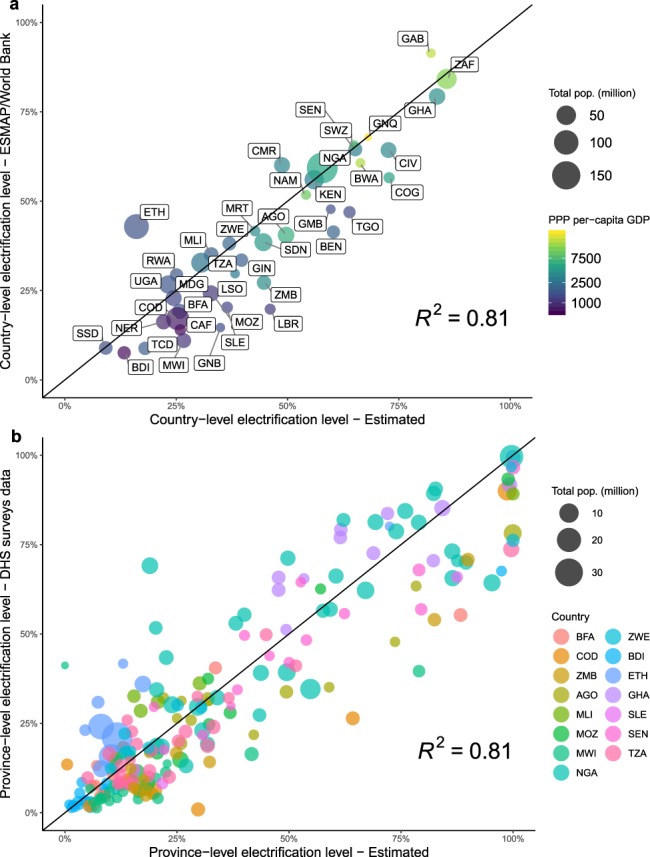
Scatterplot representing: (**a**) the national electricity access rates estimated at the pixel-level relative to values reported by ESMAP/World Bank for year 2016 (the point size is scaled to the national population and colours describe the PPP per-capita GDP of each country); (**b**) the province-level electricity access rates estimated at the pixel-level relative to USAID/DHS StatCompiler (with point size scaled to the province population and colours identifying the country of belonging). Various years are included between 2014 and 2017, depending on the survey data available for specific countries.

To assess the consistency of the estimated electrification levels, we produced a cross-sectional fit comparing the NTL estimate against the most recent official data source available, the ESMAP/World Bank *Tracking SDG 7* electrification data for the year 2016, as shown in Fig. 3a. The plot also includes a colour-legend for PPP per-capita GDP, which suggests that discrepancies tend to be larger in poorer countries. Overall, this reveals a rather consistent estimation of the heterogeneity in the data (*R*^2^ = 0.81). The remote sensing approach performs well in terms of reproducing the electrification levels - among others - in Botswana, Namibia, Tanzania, Uganda, Mali, Guinea, Madagascar, and Namibia.

The largest underestimations are found for Ethiopia (−26.8%), Cameroon (−11.3%), Gabon (−9.2%), while electrification levels are significantly overestimated vis- a-vis official figures in Liberia (+26%), Guinea Bissau (+20.2%), and Benin (+18.9%). Downward bias can be mainly attributed to - amongst other reasons - the underdetection of standalone access solutions such as solar-home-systems, or to the low level of lighting among highly distributed communities. For instance, the large discrepancy in Ethiopia could be owing to the very rapid diffusion of solar-home-systems among rural households over the last years (4 million off-grid solar devices sold between 2015 and 2017, with a penetration of 20% in 2017^61^), which might be scarcely detected by NTL data due to the inherent mismatch between solar power production and night-time light measurements in the absence of diurnal storage systems. Moreover, limited interior lighting might not be visible from outside and thus detectable using the satellite imagery.

Upward discrepancies (overestimations) are prone to be attributable to a number of factors. These encompass illegal connections (refer to^62,63^ for a recent contribution to estimation of electricity thefts), other sources of light (e.g. from the effect of gas flaring, leading to very high and diffuse radiance detected by satellites and poorly filtered by data processing algorithms), or from the presence of a large number of people without access even in areas where there is electricity (such as in urban slums and peri-urban settlements).

Finally, it was assumed that a 1-*km* pixel being lit implies that everyone in that location has access. This assumption implies that these people are in proximity to connection possibilities, i.e. remoteness is not a significant problem. At the same time, a pixel being identified as dark still allows for the possibility that some form of (standalone) electricity generation is in place. Therefore, our estimate might be interpreted best as a measure of grid-based electricity access. Crucially, the accuracy of official electrification levels and the differing survey criteria by which a household is considered electrified across countries can affect the validation. In any case, both caveats are mitigated here by the high resolution of the data, which limits potential errors of misclassifying populations as with or without access.

Overall, the validation shows that our bottom-up approach to estimate electrification levels - derived from 1-*km* grid cell level estimates - represents a generally effective method for predicting national electrification levels. In an attempt to perform a more precise validation, we collected survey-derived province-level shares of households with access to electricity from the USAID/DHS Program STATcompiler (demographic and health surveys, and disease-related surveys)^10^. This was possible for fifteen SSA countries (Angola, Burundi, Burkina Faso, DR Congo, Ethiopia, Ghana, Mali, Malawi, Mozambique, Nigeria, Senegal, Sierra Leone, Tanzania, Zambia, and Zimbabwe). It is emphasized that the the surveying period of data reported by the USAID/DHS STATcompiler often spans between two calendar years. Therefore, to produce a consistent validation, we referred to different years (between 2014 and 2017) dependent on the survey year in each country, and produce NTL-based estimates for each year. Also, USAID/DHS data are subject to smoothing processes due to the use of sampling weights (equivalent to the frequency that the sampling unit represents in the target population.), which might skew access rate estimates^10^.

Our methodology is found to be consistent with the specific electrification levels (*R*^2^ = 0.81), as shown in Fig. 3b, although a few inconsistencies exist. In particular, greater error is observed between our estimate and the USAID/DHS data for provinces with smaller populations. This suggests that the approach is generally more effective in estimating electrification in more populated provinces.

**Fig. 4:**
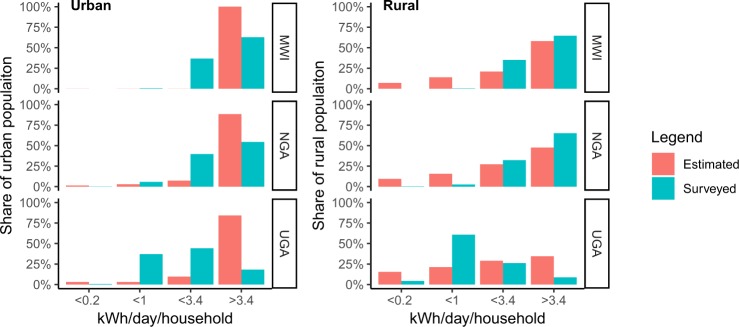
Validation of estimated consumption tiers compared to World Bank Multi-Tier framework thresholds in urban and rural areas, drawn from household surveys for selected countries with data availability.

Last, to validate the estimated electricity consumption tiers in rural and urban areas, respectively, we retrieve household survey data from the World Bank Microdata Library (http://microdata.worldbank.org). Recent data on expenditure or consumption of electricity between 2014 and 2018 are available only for three countries: Malawi (2016–17), Nigeria (2015–16), and Uganda (2013–14). Thus, it must be highlighted that the estimate is built on data from year 2018, but it could only be validated on earlier field data. In all cases, the data collected refer to consumption in the month preceding the survey. Expenditure had to be divided by reference prices to obtain power demand. For Malawi, a price of MWK 26 in rural and MWK 35 in urban areas was used^64^, for Nigeria a price of NGN 7 in rural and NGN 12 in urban areas was considered^65^, while for Uganda a price of UGX 400 in rural and UGX 550 in urban areas was adopted^66^. Price discrepancies account for cross-subsidies and monthly fees exempted from rural households with low consumption levels^67^. Continuous consumption values have then been clustered into tiers of daily consumption following the thresholds as defined by the World Bank Multi-Tier Framework (WB-MTF)^59^, with WB-MTF Tiers 4 and 5 (the two highest) aggregated to construct a consistent set of four estimated tiers. Urban and rural areas are distinguished in the surveys for each household. Figure 4 reports the results of the validation, which show a rather consistent estimation.

The main discrepancies are observed for low consumption tiers in rural areas, where our estimate predicts a relevant fraction of the electrified households to live with less that 1 KWh/day, a result which does not find robust evidence in surveys. In all likelihood, the main reason behind this discrepancy is that survey questions about electricity expenditure and consumption only target grid-connected households, and thus results underrepresent communities relying on decentralised electricity access solutions, which often also have low consumption levels. Another significant discrepancy is observed for households consuming more than 3.4 KWh/day in urban Uganda, overestimated by our data. Here, a plausible source for the discrepancy concerns the high population density characterising Kampala and the provinces surrounding Lake Victoria, resulting in greater urban-rural classification uncertainty.

### Sensitivity analysis

The analysis includes two main sources of uncertainty: the choice of the NTL noise floor, and the accuracy of the gridded population data across different years.

Supplementary Fig. 3a shows the change in the estimate in response to a ±25% change in the NTL data noise floor (set at 0.25 *μW* · *cm*^−2^ · *sr*^−1^ until 2016 and 0.35 *μW* · *cm*^−2^ · *sr*^−1^ for later years, as discussed in the Methods section) and can be interpreted a as a confidence interval for the estimate. Although the accuracy of the estimates are heterogeneous across countries for the three noise floors, a simple linear regression yields an adjusted *R*^2^ of 0.77 for the baseline, 0.76 for the +25%, and 0.78 for the for the −25%. Therefore, the overall fit does not vary substantially, and it is a country-specific question which noise floor minimizes the discrepancy with the World Bank reported electrification levels. In our analysis, for consistency purposes we keep the noise floor constant at the baseline. In advanced regional or country-level analysis, it is recommended to carry out ad-hoc sensitivity analysis on the parameter, by modifying the open-access scientific computing framework.

Supplementary Fig. 3b compares the validation obtained with LandScan and WorldPop data. In this case some countries’ reported electrification levels are better estimated when the WorldPop population data are used - despite a generally lower fit overall. However, the LandScan results in a *ceteris paribus* significantly more accurate prediction of national electrification level than the WorldPop. A simple linear regression produces a *R*^2^ of 0.77 for the LandScan and of 0.63 for the WorldPop data. Therefore, results underpin the choice of using LandScan as the default gridded population.

## Usage Notes

The dataset is in netCDF (Network Common Data Form) format, which is the standard default for gridded time-series data in the earth system modeling community. For the population without access dataset, the netCDF file features five layers, which describe years between 2014 and 2018. Each pixel contains the local count of the people estimated to live without access to electricity. For the tiers of consumption, the netCDF only contains data in pixels where people have access in year 2018. Pixel values range over integers between 1 and 4, where each refers to the tier of consumption estimated locally.

The data have a native resolution of 1-*km*, which allows for assessments at different scales. It can be analyzed without further edits, but also aggregated at the province level. Potential applications relate to the design of scenarios and the projection of future pathways of energy access and consumption in the sub-Saharan African continent. This is of particular relevance if the dataset is combined with gridded information on income and population to produce future scenarios of energy access and demand. As discussed in^68^, there is a need for improved data and representation of future energy demand in developing countries. The elaboration of such information in Integrated Assessment Models could substantially improve the understanding of the drivers, impacts, synergies and trade-offs between different development goals.

The data also have potential to be used in inequality and vulnerability assessments and scenarios. Including local-level information about electricity supply and quality offers new possibilities for assessing and correlating adaptation options at a finer spatial scale across different spheres, such as health, productivity, agricultural outcomes, and migration. Further uses include the improvement of the input data for long-term infrastructure optimization models at different spatial scales, or the assessment of national/village-level policies and the effects over the 2014–2018 period. At the same time, new NTL data are published on a monthly basis. This allows updating the dataset presented here and keeping track of progress with little delay as new data become released.

Finally, the methods and output datasets also exhibit significant potential for being used and improved through applications of machine learning and neural-network based methods for identifying patterns, clusters and dynamics. For instance, to date the Google Earth Engine platform^27^ allows for the implementation of up to ten different machine learning algorithms on geospatial data. Using those methods to detect electricity-related infrastructure^69^, such as power lines (as recently shown in^70^), power plants, and substations, may allow further improving electricity access and consumption estimates. Furthermore, the forthcoming VIIRS Black Marble VNP46 new NTL data product^71^ is likely to allow even a greater precision in the estimation of electrification levels and proxying of consumption levels thanks to less noisiness and more precise calibration of the data.

## ISA-Tab metadata file

Download metadata file.

Supplementary Information.

## Data Availability

The latest version of the code and guide for the dataset reproduction are hosted at https://github.com/giacfalk/Electrification_SSA_data. The code is available in two files: a JavaScript file to be imported into Google Earth Engine (step 1), and an R script to be run after having run the JavaScript file in Earth Engine (step 2). In order to run the Earth Engine script, it is necessary to create a Google account and register for Earth Engine via https://signup.earthengine.google.com. All the input data required for the analysis are openly available, with the exception of the LandScan gridded population dataset. To obtain access to population data, it is possible to either apply for an account to use the LandScan data at https://landscan.ornl.gov/user/apply, or use the WorldPop data, which are directly accessible through running the script in the repository. Analysis was run in R 3.4.4 using packages raster (2.8–19), ncdf4 (1.16), RnetCDF (1.9–1), googledrive (0.1.3), data.table (1.11.8), dplyr (0.7.8), plyr (1.8.4), ggplot2 (3.0.0), wbstats (0.2), ggrepel (0.8.0), sf (0.7–1), cowplot (0.9.3), reshape2 (1.4.3), rworldmap (1.3–6), rgdal (1.3–6), tidyr (0.8.3) and RColorBrewer (1.1–2). Furthermore, the checkpoint package (0.5.0) is used to provide a snapshot of and retrieve the required version of the packages and their dependencies. The average processing time for the Google Earth Engine Javascript code (to run and export assets to Google Drive) is about 10 minutes, while the R script takes around 10 minutes to install the required packages and dependencies, and an additional 10 minutes to generate the netCDF files and the figures and save them (although this depends on the specifications of the hardware used). Note that the running time of the R script is also sensitive to the internet connection speed, since the script retrieves the assets from Google Drive. The processing of satellite data and geospatial operations are performed at a 1000 m scale under a EPSG 4326 coordinate reference system.
