# Supplementary material for: A high-resolution gridded dataset to assess electrification in sub-Saharan Africa
Source: Sci Data. 2019 Jul 3;6:110. doi: 10.1038/s41597-019-0122-6 (PMC6610126; doi:10.1038/s41597-019-0122-6)
Supplement: Supplementary file 2 — Supplementary Information. [file 41597_2019_122_MOESM2_ESM.pdf]

A high-resolution gridded dataset to assess electrification in sub-Saharan Africa

Giacomo Falchetta[1,2*], Shonali Pachauri[1], Simon Parkinson[1,3], Edward Byers[1]

May 12, 2019

1. Energy Program, International Institute for Applied Systems Analysis (IIASA), Schossplatz 1, 2361, Laxenburg, Austria 2. Future Energy Program, Fondazione Eni Enrico Mattei (FEEM), Corso Magenta 63, 20123, Milan, Italy 3. Institute for Integrated Energy Systems, University of Victoria, PO BOX 3055 STN CSC, Victoria, Canada *corresponding author(s): Giacomo Falchetta (giacomo.falchetta@feem.it)

Supplementary Information

List of Figures

1 Scatterplot representing the pixel-level estimated national urban population shares against values reported by the World Bank. The point size represents the national population, and colours describe the PPP per-capita GDP of each country. Points are coloured coded on PPP per-capita GDP and scaled by population. 1

2 Distribution of non-zero radiance ($\mu W \cdot cm^{-2} \cdot sr^{-1}$) quartile values and distribution median values for all SSA countries in urban and rural areas. Wider distributions determine greater estimation uncertainty. 2

3 Sensitivity analysis for (a) country-level electrification levels (b) population datasets for country-level electrification levels estimation. 3

4 Distribution of radiance noise values over Lake Victoria in 2018 and 2014 showing (a) the increased noisiness observed in the VIIRS-DNB data and (b) the distributions once the adjustment is accounted for. 4

List of Tables

1 Population density thresholds used for urban areas definition in combination with MODIS land cover data. 1

Table 1: Population density thresholds used for urban areas definition in combination with MODIS land cover data.

Countries	$Inhab. \cdot km^{-2}$ threshold
Angola, Botswana, Gabon	> 175
Namibia, Zambia, Mauritania, Zimbabwe, Mozambique, Somalia, South Africa, Cape Verde, Swaziland	> 650
Lesotho, Comoros, Madagascar, Central African Republic, Mali, Tanzania	> 800
Gambia, Guinea Bissau, Liberia, Burkina Faso, Ivory Coast, Sierra Leone, Ghana, Guinea, Equatorial Guinea	> 1200
Ethiopia, Uganda, Burundi, Rwanda, Benin, Sudan, Eritrea	> 1500
Kenya, Malawi, DR Congo, Togo, Nigeria, Chad, Senegal, Niger, Congo	> 2500

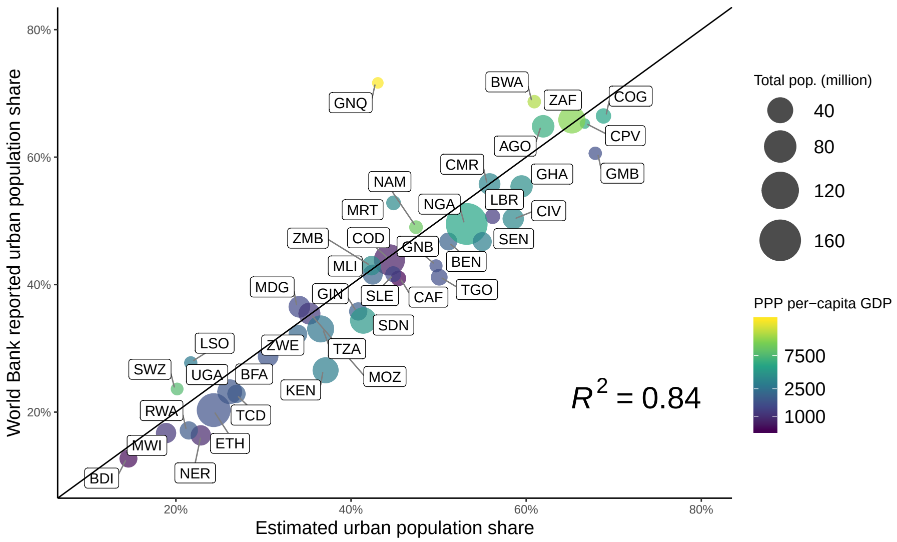

Figure 1: Scatterplot representing the pixel-level estimated national urban population shares against values reported by the World Bank. The point size represents the national population, and colours describe the PPP per-capita GDP of each country. Points are coloured coded on PPP per-capita GDP and scaled by population.

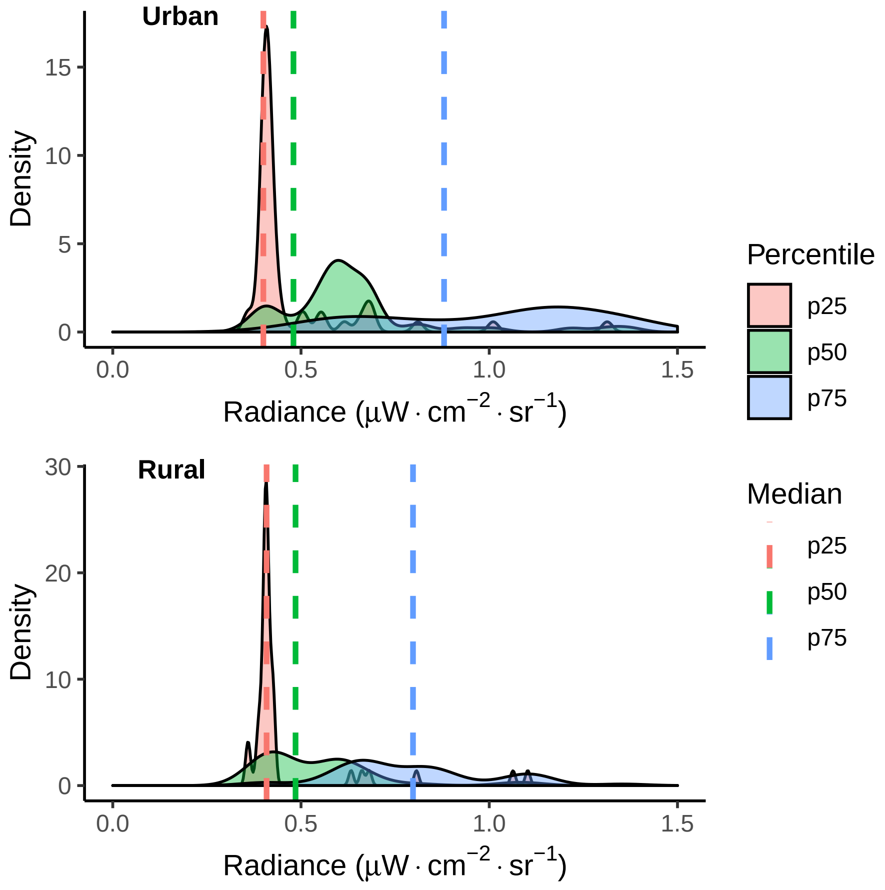

Figure 2: Distribution of non-zero radiance ($\mu W \cdot cm^{-2} \cdot sr^{-1}$) quartile values and distribution median values for all SSA countries in urban and rural areas. Wider distributions determine greater estimation uncertainty.

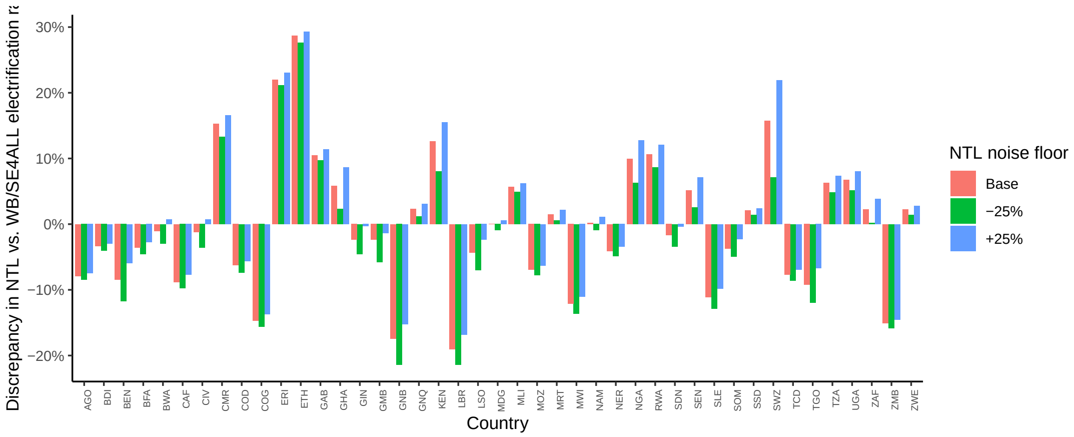

(a) Noise floor parameter

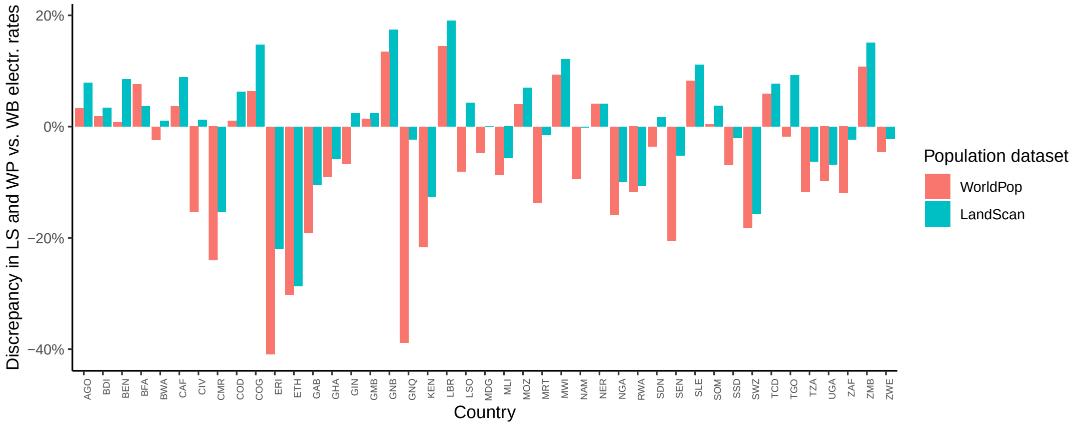

(b) Population dataset

Figure 3: Sensitivity analysis for (a) country-level electrification levels (b) population datasets for country-level electrification levels estimation.

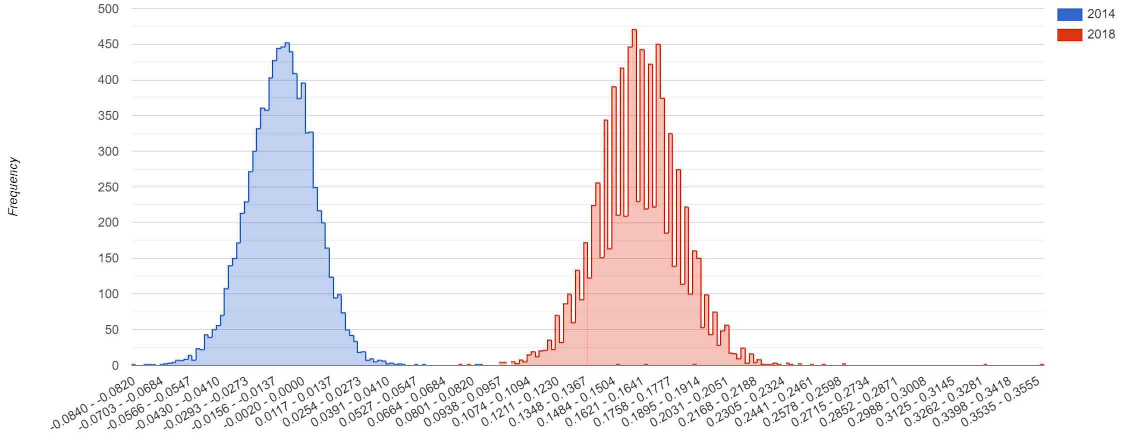

(a) Raw

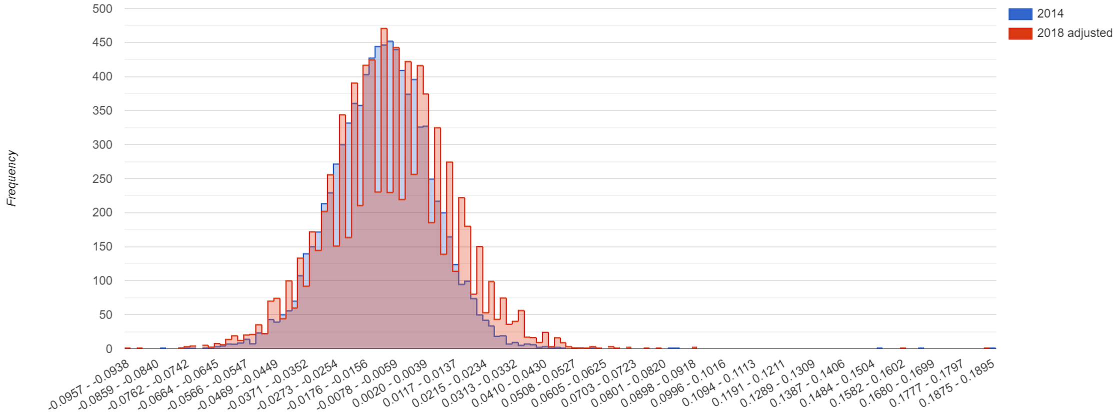

(b) Adjusted

Figure 4: Distribution of radiance noise values over Lake Victoria in 2018 and 2014 showing (a) the increased noisiness observed in the VIIRS-DNB data and (b) the distributions once the adjustment is accounted for.
